# Left Ventricular Endocardium Tracking by Fusion of Biomechanical and Deformable Models

**DOI:** 10.1155/2014/302458

**Published:** 2014-01-21

**Authors:** Hussin Ketout, Jason Gu

**Affiliations:** Electrical and Computer Engineering, Dalhousie University, 1360 Barrington Street, Halifax, NS, Canada B3J 2X4

## Abstract

This paper presents a framework for tracking left ventricular (LV) endocardium through 2D echocardiography image sequence. The framework is based on fusion of biomechanical (BM) model of the heart with the parametric deformable model. The BM model constitutive equation consists of passive and active strain energy functions. The deformations of the LV are obtained by solving the constitutive equations using ABAQUS FEM in each frame in the cardiac cycle. The strain energy functions are defined in two user subroutines for active and passive phases. Average fusion technique is used to fuse the BM and deformable model contours. Experimental results are conducted to verify the detected contours and the results are evaluated by comparing themto a created gold standard. The results and the evaluation proved that the framework has the tremendous potential to track and segment the LV through the whole cardiac cycle.

## 1. Introduction

Echocardiography is an important imaging modality that enables the cardiologist to evaluate the structure and functions of the heart. Because of noninvasive characteristics, low cost, and being nonionizing radiation, echocardiography has been largely applied in the evaluation of cardiac function. One of the most important applications of echocardiography is in determining systolic and diastolic ventricular volumes of the patient, both of which are used to calculate the left ventricular ejection fraction, muscle contraction ratio of cardiac cavities, local ejection fraction, myocardial thickness, and the ventricle mass [1]. To calculate the above-mentioned parameters, the cardiac muscle contour on the echocardiography image needs to be identified. The border detection process simplifies image analysis and greatly reduces the amount of data which needs to be processed, while preserving the structural information about the contours of the object under study [2].

However, in clinical practice, this task still relies on manual outlining. Manual outlining of these borders is slow, time consuming, and tedious task. Moreover, the resulting outlines vary between different observers and suffer from a subjective bias [3].

Automatic LV border detection and tracking over the cardiac cycle in echocardiographic image sequences remain open and a challenging problem due to many difficulties related to the heart and its dynamics and other difficulties related to the echocardiography ultrasound machine.

Echocardiography has a poor image quality and resolution with various image artifacts like speckle, shadowing, and side lobes [3]. The images of echocardiography suffer from signal dropout. This dropout in the echocardiography signal makes part of the LV invisible which yields an open contour [3]. Moreover, the relation between the physical property of the monitored tissue and the intensity of the pixels cannot be described in a simple way [4]. Due to the highly anisotropic information of the 2D echocardiography images and the mentioned artifacts, the locations of the real contour do not always correspond to the locations of the strong image features like strongest edges. Relaying on the strongest image features will not lead to the detection of the desired contour as traced by cardiologists [4]. Another challenge is the Gray level intensity variability and low signal to noise ratio [3, 5].

The difficulties and challenges that relate to the heart which impede the LV detection of LV boundaries are.

 (i) The heart is a highly deformable object with a wide range motion.
 (ii) The papillary muscle is impeding the detection of the real LV boundary.
 (iii) The mitral valve also is one of the artifacts and causes a problem for the detecting the LV boundaries.

To tackle the challenges and difficulties due to echocardiographic images and due to LV complex motion, an averaging framework is employed which is based on fusing the parametric deformable and BM models computed contours. It has been found that the different contour detectors used for a special detection task usually complement each other with respect to the information extracted from the patterns to be classified [4]. As a result, combining different contour detectors in an efficient way, is expected to achieve better contour detection results than any single contour detector, and hence produce incremental gains in overall performance.

The biomechanical model is used to mimic the mechanics of the heart muscles specifically myocardium. This particular model is centered on using finite deformation elasticity technique. Once the modeling is done using this technique, the subsequent functional is solved using FEM. The accurate model of myocardial mechanics can simulate the heart wall motion during the diastolic and systolic stages of the cardiac cycle [6, 7].

Instead of depending only on the echocardiographic images to detect the LV boundaries, a FEM model for mimicking the LV movement during a cardiac cycle is developed to obtain the LV boundaries from the functions of transversely isotropic material biomechanical model introduced by Lin and Yin [8].

The paper is organized as follows.  Section 2 presents the tracking framework.  Section 3 introduces the BM model along with the constitutive equations that are used in our work. The boundary conditions and the ABAQUS solver are illustrated also in detail.  Section 4 is about the parametric deformable model.  Section 5 gives the details of the averaging fusion technique. Experimental results are given in Section 6. Evaluation of the results is provided in Section 7. The discussion and the analysis of the results are given in Section 8. The future work is mentioned in Section 9. The paper's conclusion is given in Section 10.

## 2. Tracking Framework

**Figure 1:**
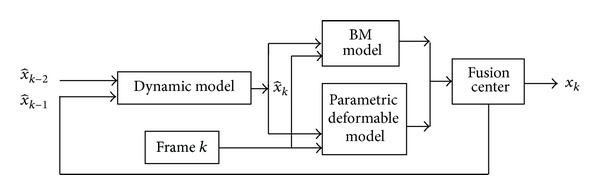
LV endocardium contour tracking framework.

The endocardium contour tracking framework for 2D echocardiographic image sequence is shown in Figure 1. The user needs to click inside the ROI and the level set [10] will be used to provide the initial contour *x*_0_. The predicted state of the LV contour will be provided by the dynamic model. To enable modeling of motion in addition to position, second-order dynamical model is used and given as follows [11, 12]:

(1) $$\begin{array}{c} {\hat{x}}_{k}=A_{1}{\hat{x}}_{k-1}+A_{2}{\hat{x}}_{k-2}+\left(I-A_{2}-A_{1}\right)\overset{-}{x}, \end{array}$$

where $\overset{-}{x}$ is the mean shape of the LV, ${\hat{x}}_{k}$ is the LV contour at time step *k*, and ${\hat{x}}_{k-1}$ is the LV contour at time step *k* − 1.

## 3. BM Model Endocardial Contour Detection

Unlike the imaging based techniques, the BM model endocardial contour detection is based on finding the LV deformations by using the heart model and solving the model equations using ABAQUS FEM. By using the BM model and getting LV deformations through the FEM, this technique enables us to avoid and tackle all the challenges and difficulties of segmenting and tracking the LV through echocardiography image sequence that was mentioned in the introduction. The following are the parts of BM model solution.

### 3.1. Constitutive Equations

A constitutive equation is a mathematical model that characterizes the relationship between stress and deformation. To mimic the LV movement, our work is based on the Lin-Yin model [8] for the constitutive equations based on hyperelastic material theory. In this model, the strain energy functional *W* is divided into two components: one is the passive (*W*_pass_) and the other is the active (*W*_active_). Lin and Yin used an exponential function form for the passive strain energy function given as follows:

(2) $$\begin{array}{c} W_{\text{pass}}=c_{1}\left(e^{Q}-1\right),\\ Q=c_{2}{\left(I_{1}-3\right)}^{2}+c_{3}\left(I_{1}-3\right)\left(I_{4}-1\right)+c_{4}{\left(I_{4}-1\right)}^{2}, \end{array}$$

where *c*_1_, *c*_2_, *c*_3_, and *c*_4_ are the material properties parameters that determined experimentally.

The active strain energy function is given in the polynomial form as follows [8]:

(3) Wactive=c5+c6(I1−3)(I4−1)+c7(I1−3)2 +c8(I4−1)2+c9(I1−3)+c10(I4−1).

Also, *c*_5_, *c*_6_, *c*_7_, *c*_8_, *c*_9_, and *c*_10_ are the material properties parameters that are determined experimentally [8]. *I*_1_ and *I*_4_ are the invariants of right Cauchy deformation tensor and they are given by the following equations:

(4) $$\begin{array}{c} I_{1}=\mathrm{tr}C_{ij}=C_{11}+C_{22}+C_{33},\\ {I_{4}}^{2}=N_{i}C_{ij}N_{j}\text{.} \end{array}$$

### 3.2. BM Model Endocardial Contour Detector Framework

**Figure 2:**
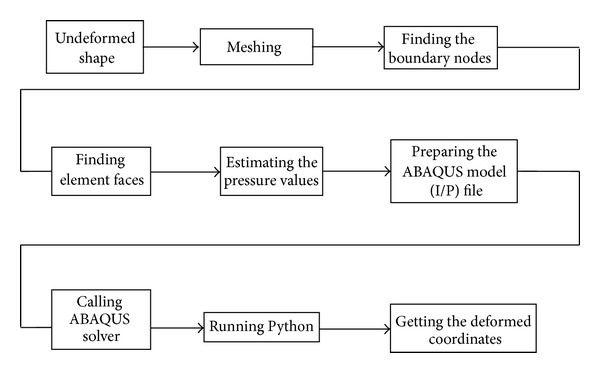
FEM solution block diagram.

**Figure 3:**
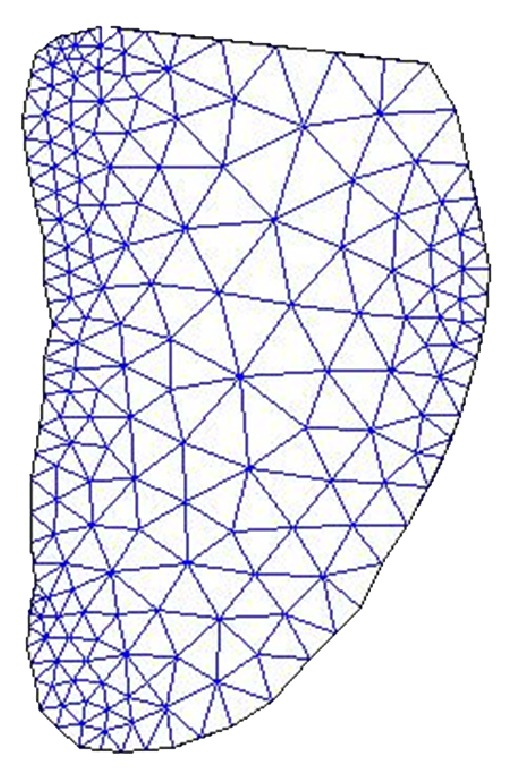
Meshing the LV into M domain cells.

**Figure 4:**
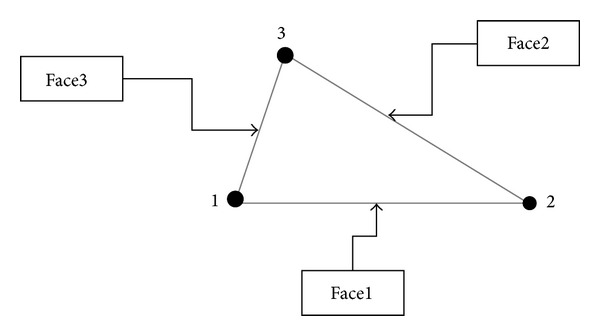
Identifying triangle element's faces.

The BM model endocardial contour detector for 2D echocardiographic image sequence is shown in Figure 2. The undeformed shape is obtained by using previous frame in the cardiac cycle. The meshing of LV domain is obtained by dividing the domain into triangles as shown in Figure 3. After meshing the LV domain, the next step is finding and identifying all the nodes at the boundary of the LV. This step is necessary to find the nodes where the boundary conditions should be applied. After finding the nodes that form the boundaries of the LV, the elements which relate to the boundary nodes will be identified. After knowing the boundary elements, the faces of each boundary element must be recognized. Each triangle element has three faces. These faces are ordered in anticlockwise as shown in Figure 4. Then, next the face of the triangle element at the boundary should be finding and identified as Face1, Face2, or Face3. After identifying all the faces at the boundary of the LV, the same faces will be grouped together in one group. After that, all the groups will be combined together to form one SURFACE. This SURFACE is where the pressure should be applied during the systole and diastole stages of the cardiac cycle.

### 3.3. Boundary Conditions

From the heart anatomy, LV is bridled by the atria, RV (right ventricle), and the aorta. The quantitative information of the boundary conditions between these parts of the heart is unknown. To prevent rigid body motion of the left ventricle during the deformation calculation, the basal plane motion should be suppressed.

The load applied to the endocardial SURFACE is the blood pressure and the blood pressure in human heart depends on time and location. The fluid dynamics of the blood pressure in the left ventricle should be taken into account to estimate the spatial distribution of the blood pressure as a function in time. During LV contraction, the pressure gradients are very small compared to the absolute pressure. From this, we can assume a uniform parabolic distribution of the pressure along the endocardial SURFACE of the LV.

From tracking of the cardiac cycle, the duration of systole phase lasts for 16 frames starting at the QRS ECG signal. The pressure starts rising up to 80 mmHg and reaches the peak of 120 mmHg and down to 80 mmHg at the end of systole stage.

In the literature [13], the blood pressure of the left ventricle varying with time is simulated as follows:

(5) $$\begin{array}{c} t=0.055+k*0.0090625,\\ P=-944.38t^{2}+245.54t,\;0\leq t\leq 0.2. \end{array}$$

The total duration of time is divided over the 16 frames which covers the systole stage (*k* represents the frame number). According to the frame number *k*, the value of *t* will be calculated using first equation of (5). The pressure value will be calculated from the second equation of (5). The minimum pressure will be 10.666 Kpa which corresponds to 80 mmHg while the maximum pressure value is 15.96 Kpa which corresponds to 120 mmHg. These values are the normal systolic blood pressure for an intact heart.

In diastole stage, the pressure is approximately fixed and a value of 5 Kpa is used as indicated in the pressure curve in the cardiac cycle diagram.

### 3.4. ABAQUS Solver

For each frame of the echocardiographic image sequence, ABAQUS model (input file) is prepared by MATLAB script that identifies the entire model and history data which are saved in the input file and run by ABAQUS to estimate the deformations of the LV at that moment in the cardiac cycle. Beside, the input file, two user subroutines are prepared and used in the framework. To identify the biomechanical model to ABAQUS, both the passive and active strain energy functions should be declared in a specific user subroutine in ABAQUS called UANISOHYPER_STRAIN.F [14, 15]. ABAQUS will be called from MATLAB script and it will run in background mode.

After the completion of the ABAQUS model, a Python program will be called from MATLAB script to read the results from ODB file (Output Data Base) [16, 17].

The Python program will read the deformations (displacements) at each boundary node and add it to the original coordinates to find the deformed contour. After the completion of these calculations, the MATLAB running will continue to find the LV deformed contour using parametric deformable model and then fuse it with BM estimated contour as illustrated in the following sections.

## 4. Parametric Deformable Model

Kass et al. [18] introduced the concept of active contour models (ACM), or Snake in his paper “Snakes: active contour models.” Snakes are used in the area of image processing to detect the object boundaries. Snake is modeled as parametric curve that evolves into a position where its energy functional is minimized. The position of the Snake is given by the parametric curve *C*(*s*) = [*x*(*s*), *y*(*s*)] with *s* = [0, 1]. Kass et al. introduced the following energy functional for the Snake:

(6) $$\begin{array}{c} E_{\text{Total}}=\overset{1}{\underset{0}{\int }}\left(E_{1}\left(c\left(s\right)\right)+E_{2}\left(c\left(s\right)\right)\right)ds, \end{array}$$

where

(7) $$\begin{array}{c} E_{1}=\overset{1}{\underset{0}{\int }}\left(\alpha {||c^{\prime }\left(s\right)||}^{2}+\beta {||c^{\prime \prime }\left(s\right)||}^{2}\right)ds,\\ E_{2}=\overset{1}{\underset{0}{\int }}P\left(c\left(s\right)\right)ds. \end{array}$$

*E*_1_ is the internal energy term and *E*_2_ represents the external energy term. The first term in the internal energy represents the elasticity and the second term represents the curvature. The influence of the two terms is controlled by the parameters *α* and *β*, respectively. The external energy (image energy) attracts the Snake to the boundaries of the object in the images. The image energy here will be defined as follows:

(8) $$\begin{array}{c} E_{2}=-{||\nabla I\left(x,y\right)||}^{2}, \end{array}$$

where *I* is the image function. Following this, the Snake function will be minimized in the position with high gradient values.

parametric deformable models are used at this stage due to the way that they represent their curves with a set of control points in the same manner that we used to represent the contour curve in the BM model. BM model represents the curve as a set of nodes. This similarity enables us to use point to point mapping to fuse both contours of Snake and BM model as illustrated in the next section.

## 5. Fusion Using Averaging Technique

Averaging fusion technique is based on establishing one-to-one correspondence between the control points of the Snake and BM model contours [9]. The first step in the averaging fusion technique is to compute the average contour *C*_avg_ using the following formula:

(9) $$\begin{array}{c} y_{i}=\frac{1}{M}\overset{M}{\underset{j=1}{\sum }}x_{ji}. \end{array}$$

After getting the average curve, at each point on the *C*_avg_ curve, a normal to the curve is calculated. An efficient method is used to compute the normal given by [19] based on a 2 × 2 scatter matrix given as follows:

(10) A=[a11a12a  21  a22].

The matrix *A* is given by following formula:

(11) A=1∑i[(xi−x−)2+(yi−y−)2]×[∑i(xi−x−)2∑i(xi−x−)(yi−y−)∑i(xi−x−)(yi−y−)∑i(yi−y−)2].

First, the eigenvalues of the matrix are calculated and represented as *α*_*M*_ and *α*_*m*_. After that the orthonormal eigenvectors (*a*_*M*_, *a*_*m*_) are calculated using the following formula:

(12) $$\begin{array}{c} a_{M}=\frac{\left(a_{12},\alpha_{M}-a_{11}\right)}{\sqrt{a_{12}^{2}+{\left(\alpha_{M}-a_{11}\right)}^{2}}},\\ a_{m}=\frac{\left(a_{11}-\alpha_{m},a_{12}\right)}{\sqrt{a_{12}^{2}+{\left(\alpha_{M}-a_{11}\right)}^{2}}}. \end{array}$$

**Figure 5:**
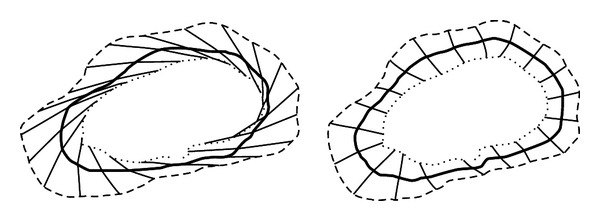
Averaging steps, firstly, finding the correspondence between each control point in both contours and lastly, the averaged contour with the bold line [9].

After computing the normal, the intersection between the normal vector with BM and Snake contours will be computed. These intersection points give us a new correspondence between both contours which will be averaged again using (9). This procedure will be iterated until we find that there is no change in the computed averaged points of both contours. Usually the iteration process takes 5 iterations to compute the final averaged contour [9].  Figure 5 illustrates the steps of the averaging technique.

## 6. Experimental Results

**Figure 6:**
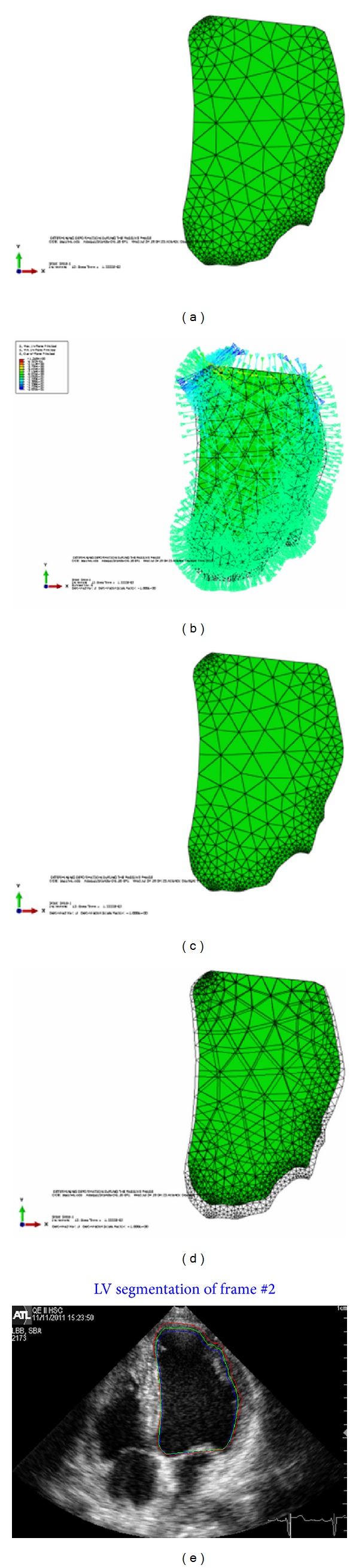
(a) Undeformed shape. (b) Load applied to the undeformed shape of LV at contraction phase. (c) Deformed shape. (d) Superposition of the undeformed and deformed shapes of LV. (e) Plotting all contours in the ultrasound image, blue for BM model, red for Snake, and green for the fused contour.

Our implemented framework is used to estimate the deformations of the left ventricle at each frame of the cardiac cycle for the 2D echocardiographic image sequence. The tracking starts at the QRS of the ECG signal which marks the end of diastole and starting of the systole phase. The BM model estimates the LV deformation at each control point (nodes at the boundary), adding these calculated deformations to the previous contour resulting in the final contour. The FEM will run for a certain time with the computed pressure (load) at each frame of the cardiac cycle. The BM model estimated contour is fused with the Snake contour to get the endocardial contour of the LV. Some experimental results are conducted to verify the framework. In Figure 6, a sample of four-chamber view is used to test the framework. The first image, Figure 6(a), shows the undeformed shape of the LV. The second image, Figure 6(b), shows the pressure applied to this frame. The load is positive in the contraction phase and applied at all the faces of the elements at the boundary of the LV. This positive pressure will let the LV boundaries to contract and the volume of the LV will be less in the deformed shape. No pressure is applied at the base and this surface should be kept fixed to avoid rigid body motion.

Starting from the undeformed shape and applying the pressure to the endocardial SURFACE, the LV will contract and the deformed shape will be as shown in the third image Figure 6(c). The forth image, Figure 6(d) shows superimposing the undeformed and deformed shapes of the LV before and after contraction. As in the real LV, more contraction will occur at the apex and lateral wall while less contraction will occur at the septal.  Figure 6(e) shows plotting of the all estimated contours, the BM model, Snake, and the fused contour in the current frame of 2D echocardiographic image sequence.

**Figure 7:**
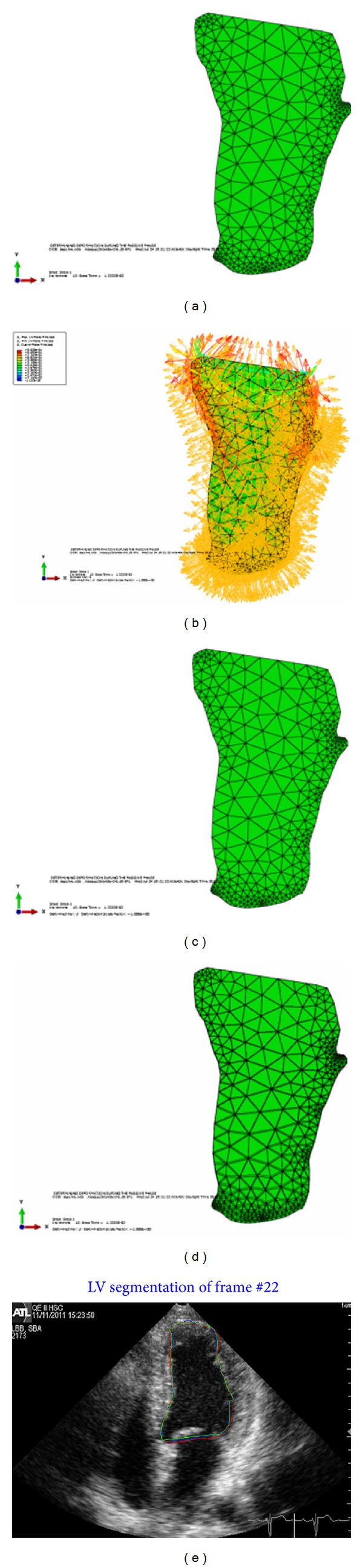
(a) Undeformed shape. (b) Load applied to the undeformed shape of LV at the relaxation phase. (c) Deformed shape. (d) Superposition of the undeformed and deformed shapes of LV. (e) Plotting all contours in the ultrasound image, blue for BM model, red for Snake, and green for the fused contour.

Next figure shows the results of applying the framework to a certain image in the passive phase of the cardiac cycle.  Figure 7(a) shows the undeformed shape before applying the load.

**Figure 8:**
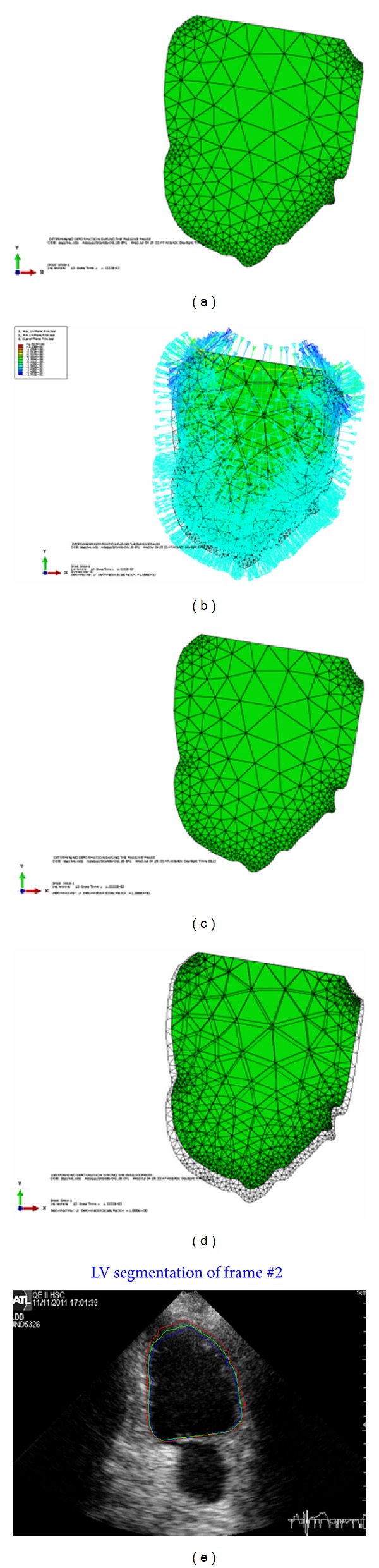
(a) Undeformed shape. (b) Load applied to the undeformed shape of LV at the contraction phase. (c) Deformed shape. (d) Superposition of the undeformed and deformed shapes of LV. (e) Plotting all contours in the ultrasound image, blue for BM model, red for Snake, and green for the fused contour.

**Figure 9:**
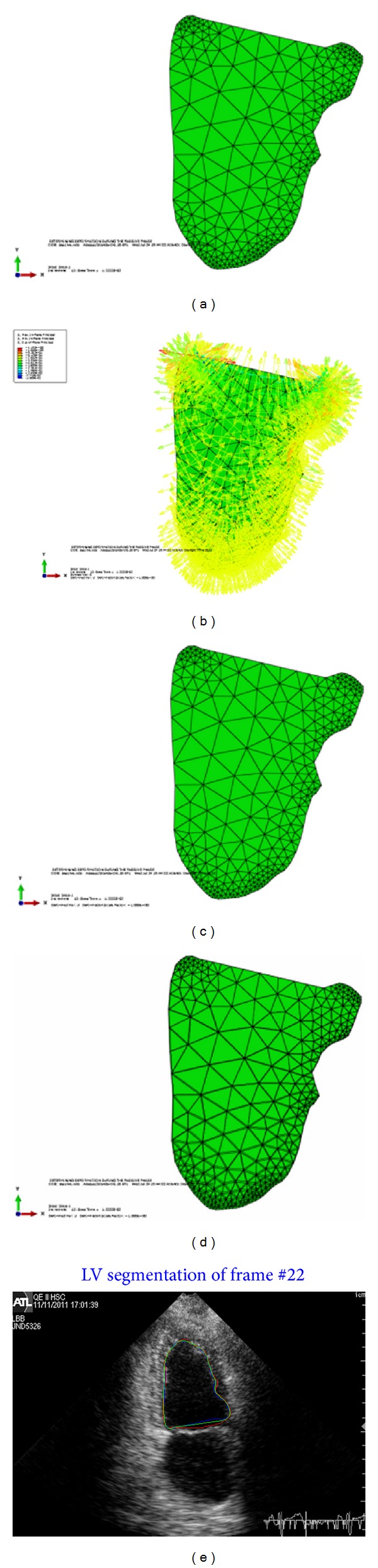
(a) Undeformed shape. (b) Load applied to the undeformed shape of LV at the relaxation phase. (c) Deformed shape. (d) Superposition of the undeformed and deformed shapes of LV. (e) Plotting all contours in the ultrasound image, blue for BM model, red for Snake, and green for the fused contour.

Figure 7(b) shows the load applied to the undeformed shape of the LV. As shown in the figure, the pressure in this case is negative (arrows are pointing outward) to let the LV boundaries expand to simulate the relaxation of the LV muscles in the passive phase. As mentioned in the contraction results, no pressure was applied at the base; only pressure was applied to the faces of the elements at the LV boundaries. The third image, Figure 7(c), shows the LV shape after applying the load. The undeformed and the deformed shapes are superimposed together to show the amount of deformation that happened after applying the load as shown in Figure 7(d). In the last image of Figure 7(e), the LV computed contours are plotted in the 2D echocardiographic image. Two-chamber view results are given in the next two figures to show the performance of the framework.  Figure 8 shows the estimation of the LV contour in the active phase while Figure 9 shows the results in passive phase.

**Figure 10:**
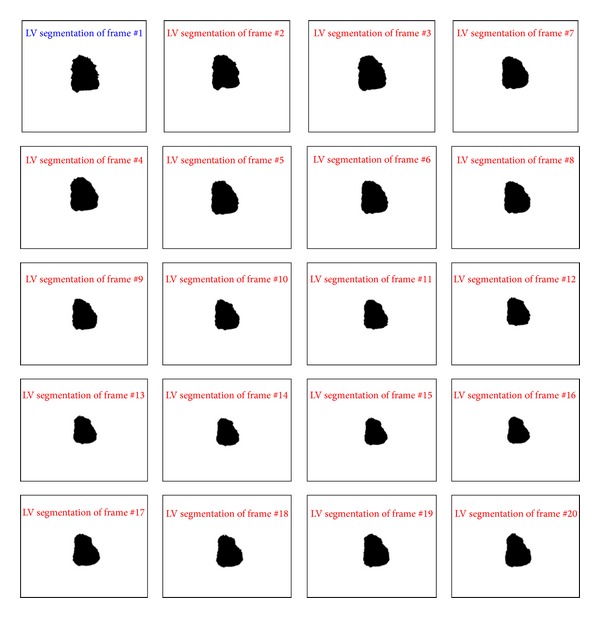
Segmentation of 20 frames of two-chamber view starting from the end of diastole to the end of systole.

Figure 10 shows the segmentation of LV area from the sequence of two-chamber view. The sequence starts from the QRS signal that marks the end of diastole stage and beginning of the systole stage. 20 frames are used to show the deformations of the LV and its area at each frame and the robustness of the framework to extract the exact area of the LV.

## 7. Evaluation of the Results

**Figure 11:**
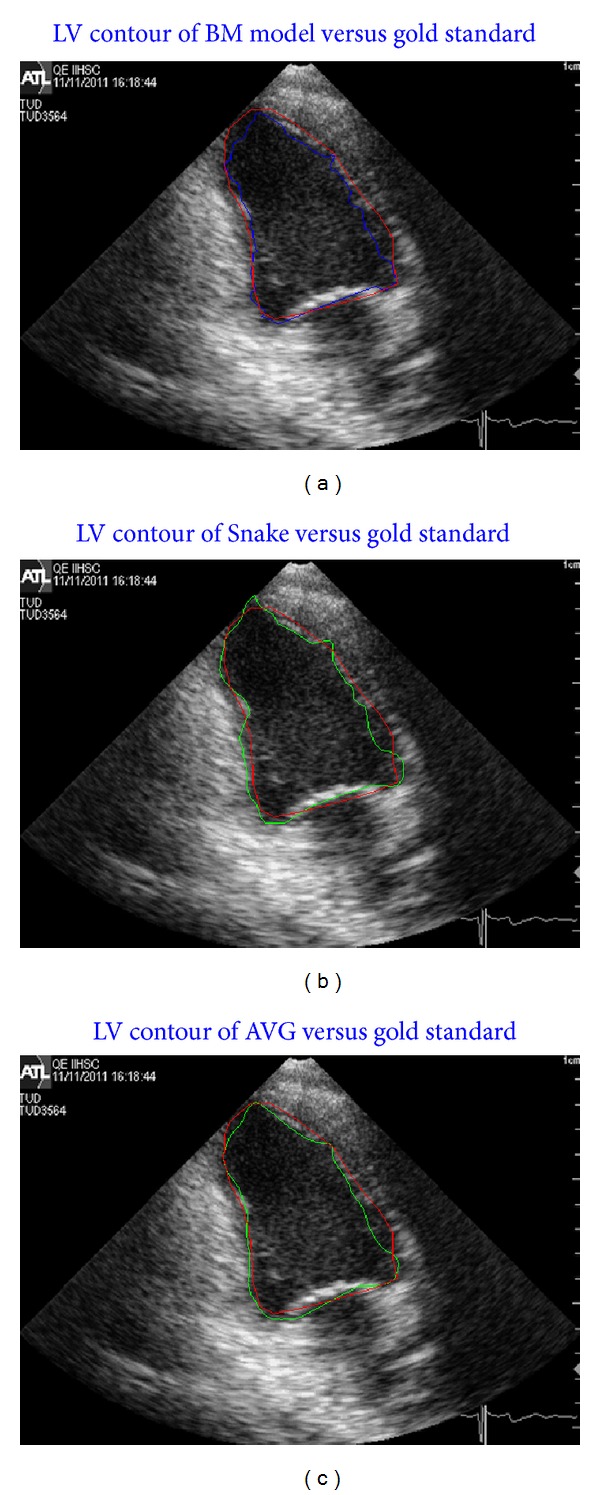
(a) Plotting the BM contour (blue line) versus gold standard contour (red line), (b) Snake contour (green line) versus gold standard contour (red line), and (c) AVG (green line) contour versus their gold standard (red line) at the end of the diastole stage.

The results of the framework are evaluated by comparing them to a gold standard created from three manually plotted contours traced by three cardiologists. The procedure of creating the gold standard is given in [9] is done by taking the average of the three curves after establishing the correspondence between the points in each curve. Ten samples are used to create the gold standard. Average perpendicular distance (APD) is used as an error metric to measure the closeness of the estimated contours to the gold standard.  Figure 11 shows samples of comparing the BM model, Snake, and the fused computed contours with the created gold standard contour.  Figure 11(a) shows the plotting of the BM estimated contour versus the gold standard of the same frame which is at the end of diastole stage. The computed contour matches the gold standard without leaking outside the boundaries at parts of the contour that has signal drop out.  Figure 11(b) shows the plotting of the Snake on the same frame versus the gold standard. The computed contour has leakage outside the real boundaries of the LV due to the signal drop out. Due to the open contour, Snake does not find the edges at that part of the contour. Unlike the Snake, BM does not depend on the ultrasound image to estimate the contour. As shown in Figure 11(a), this shortcoming of the ultrasound can be avoided by using BM model. The fusing of the BM and Snake contours is shown in Figure 11(c). The fused contour shows more closeness to the gold standard than both BM and Snake contours. Also, more smoothness with rejecting the overshoots has been done by fusing both contours.

**Figure 12:**
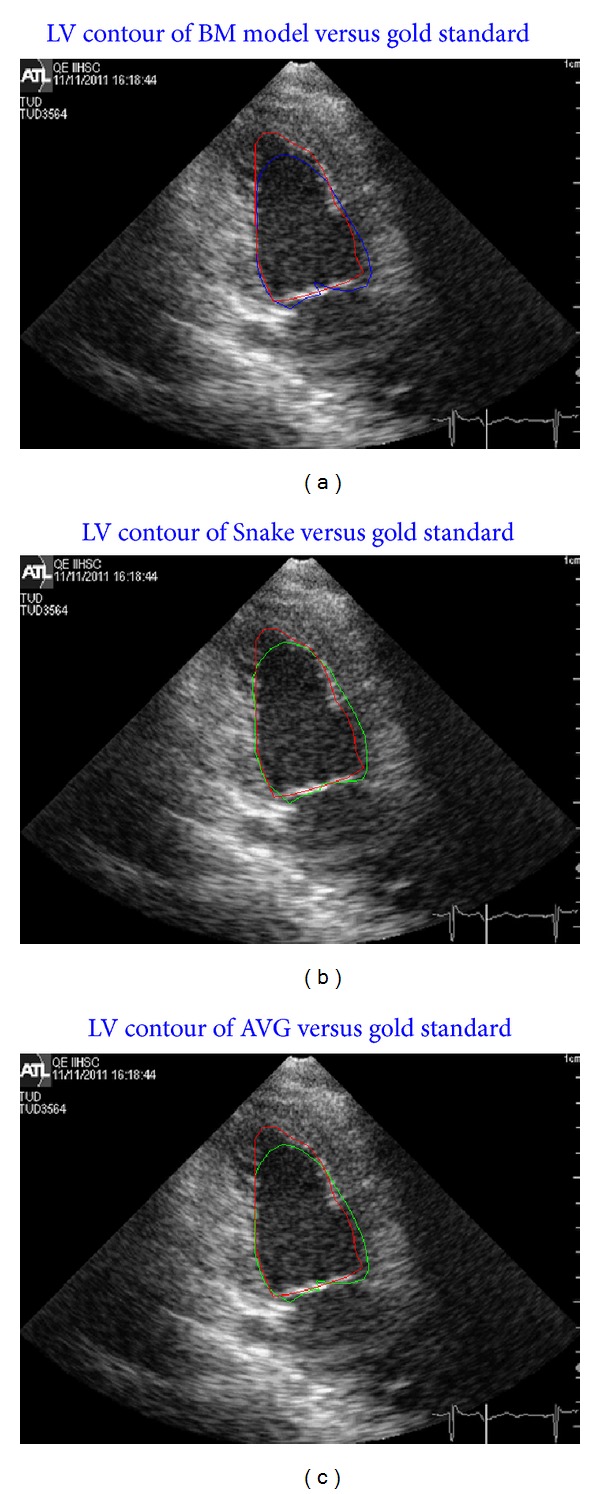
(a) Plotting the BM contour (blue line) versus gold standard contour (red line), (b) Snake contour (green line) versus gold standard contour (red line), and (c) AVG (green line) contour versus gold standard (red line) at the end of the systole stage.

In the same manner, Figure 12 shows the comparison of the three contours with the gold standard at the end of the systole stage.

**Figure 13:**
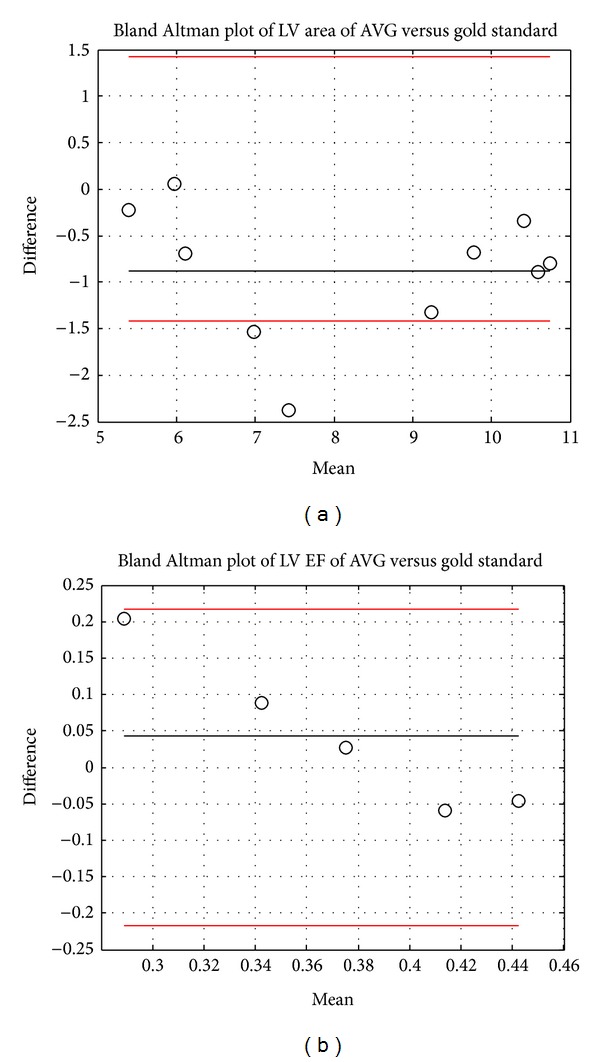
Bland Altman plot for LV computed (a) area and (b) EF.

In Figure 13, the computed area and ejection fraction (EF) values are compared to the gold standard.

## 8. Discussion and Analysis of the Results

By incorporating BM model in the averaging fusion technique framework and from the experimental results and the evaluation, the framework achieves high robustness and stability overall the samples during the cardiac cycle. The computed contours show a high closeness to the gold standard. BM model plays a dominant rule in the framework by providing the concrete base that the framework stands on during the cardiac cycle. The BM model works independently from the ultrasound images and can provide accurate detection to the LV boundaries where the deformable models fail to do so. BM model keeps the deformable models inside the ROI by overcoming the difficulties of twisting and rotation of the LV and preventing the deformable models from leaking outside the ROI when there are missing parts of LV boundaries or signal drop out.

By employing averaging fusion techniques, we ensure that the fused contour is close to the boundaries of the LV by removing the outliers in the deformable models and modifying the BM model contour. The fused contour and the dynamic model provide robustness and accurate starting point by initializing the current frame with closeness contour to the desired one. This accurate initialization ensures the quality of the contour detection and reduces the required running time for the deformable models by reducing the number of iterations that the deformable models required.

The constitutive equations of the BM model resemble and simulate the intact heart. From the experimental results and the evaluation, the BM model and the framework overall have accurate detection of the contour, area and ejection fraction for the cases in which the heart is normal or suffers from fewer complications and abnormality. In the cases where the patient has severe heart abnormality, the heart of patient does not contract as the normal (dysfunction case) and the stroke volume is less than normal value too. In this situation, the BM contour will not match exactly the real contour and it will affect the accuracy of the fused contour.

From the APD data and the statistical analysis, the fused contour scores the highest accurate results to be the closet contour to the gold standard with 1.313 ± 0.0206 mm as shown in Table 1. Also, the fused contour has the outstanding results in computing the area and ejection fraction values. In calculating the ejection fraction, all the computed values are located in acceptance range of the Bland and Altman plot as shown in Figure 13.

## 9. Future Work

Based on the LV tracking performance in 2D echocardiography image sequence, the framework will be extended for 3D echocardiography using 3D BM and deformable models.

## 10. Conclusion

A novel approach to integrate a left ventricular BM model within the framework of FEM has been presented and fused the estimated contour with a parametric deformable model. LV deformations through 2D echocardiographic image sequence are tracked for the whole cardiac cycle by this framework to tackle the challenges and difficulties of the ultrasound images and the heart. The BM model uses the constitutive equations of both the passive and active strain energy functions to simulate the myocardial tissue movement. Nonlinear deformations are estimated by solving the constitutive equations using ABAQUS FEA. Averaging fusion technique is used to fuse the BM and the deformable model contours. The experimental results are conducted and evaluated with a created gold standard. According to the experimental results and the evaluation, this approach shows a tremendous potential to track the LV endocardium during the cardiac cycle and tackle the difficulties and challenges due to 2D echocardiography and the heart.

## Figures and Tables

**Table 1:** Computing the mean of the measured APDs.

Method	Mean (mm)
BM model	1.566
Snake	1.515
AVG	1.313

## References

[1] Chan S, Sainarayanan G (2006). Fuzzy-based boundary enhancement for echocardiogram using local image characteristics. *Malaysian Journal of Computer Science*.

[2] Ketout H, Gu J, Horne G (2011). Improved dempster and shafer theory to fuse fuzzy inference system, neural networks and CNN endocardial edge detection. *The Mediterranean Journal of Electronics and Communications*.

[3] Sainarayanan G, Mal Murugan N, Chan S A novel method for echocardiogram boundary detection using adaptive neuro-fuzzy systems.

[4] Bosch JG, Mitchell SC, Lelieveldt BPF (2002). Automatic segmentation of echocardiographic sequences by active appearance motion models. *IEEE Transactions on Medical Imaging*.

[5] Zhang LF, Geiser EA (1984). An effective algorithm for extracting serial endocardial borders from 2-dimensional echocardiograms. *IEEE Transactions on Biomedical Engineering*.

[6] Nielsen PMF, Le Grice IJ, Smaill BH, Hunter PJ (1991). Mathematical model of geometry and fibrous structure of the heart. *American Journal of Physiology: Heart and Circulatory Physiology*.

[7] Nash MP, Hunter PJ (2000). Computational mechanics of the heart. *Journal of Elasticity*.

[8] Lin DHS, Yin FCP (1998). A multiaxial constitutive law for mammalian left ventricular myocardium in steady-state barium contracture or tetanus. *Journal of Biomechanical Engineering*.

[9] Chalana V, Kim Y (1997). A methodology for evaluation of boundary detection algorithms on medical images. *IEEE Transactions on Medical Imaging*.

[10] Li C, Xu C, Gui C, Fox MD Level set evolution without re-initialization: a new variational formulation.

[11] Blake A, Isard M (2000). *Active Contours*.

[12] Blake A, Isard M, Reynard D (1995). Learning to track the visual motion of contours. *Artificial Intelligence*.

[13] Dorri F, Niederer PF, Lunkenheimer PP (2006). A finite element model of the human left ventricular systole. *Computer Methods in Biomechanics and Biomedical Engineering*.

[18] Kass M, Witkin A, Terzopoulos D (1988). Snakes: active contour models. *International Journal of Computer Vision*.

[19] Anderson IM, Bezdek JC (1984). Curvature and tangential deflection of discrete arcs: a theory based on the commutator of scatter matrix Pairs and its application to vertex detection in planar shape data. *IEEE Transactions on Pattern Analysis and Machine Intelligence*.

